# Progressive White Matter Microstructure Damage in Male Chronic Heroin Dependent Individuals: A DTI and TBSS Study

**DOI:** 10.1371/journal.pone.0063212

**Published:** 2013-05-01

**Authors:** Yingwei Qiu, Guihua Jiang, Huanhuan Su, Xiaofei Lv, Xuelin Zhang, Junzhang Tian, Fuzhen Zhuo

**Affiliations:** 1 Department of Medical Imaging, Guangdong No.2 Provincial People’s Hospital, Guangzhou, People’s Republic of China; 2 Departments of Medical Imaging and Interventional Radiology, Cancer Center, Sun Yat-Sen University, Guangzhou, People’s Republic of China; 3 Department of Radiology, DongGuan KangHua Hospital, Sun Yat-Sen University, Donggua, People’s Republic of China; 4 Addiction Medicine Division, Guangdong No.2 Provincial People’s Hospital, Guangzhou, People’s Republic of China; University of Jaén, Spain

## Abstract

**Background:**

To investigate the WM microstructure deficits in heroin dependent individuals (HDIs) with different length of heroin dependence, and to investigate whether these WM deficits can be related to the duration of heroin use and to decision-making deficits in HDIs.

**Methodology/Principal Findings:**

Thirty-six HDIs [including eighteen sHDIs (duration of heroin dependent is less than 10 years) and eighteen lHDIs (duration of dependent is between 10∼20 years)] and sixteen healthy controls participated in this study. Whole brain voxel-wise analysis of fractional anisotropy (FA), mean diffusivity (MD), axial diffusivity (Da) and radial diffusivity (Dr) were performed by tract-based spatial statistics (TBSS) to localize abnormal WM regions among groups. TBSS demonstrated that sHDIs had significantly lower FA than controls in right orbito-frontal WM, bilateral temporal WM and right parietal WM. The lHDIs had significantly lower FA throughout the brain compared with the controls and sHDIs. The lHDIs had significantly lower Da than controls in bilateral inferior frontaloccipital fasciculus, bilateral splenium of corpus callosum, left inferior longitudinal fasciculus, and had significantly higher Dr than controls in bilateral uncinatus fasciculus, bilateral inferior frontaloccipital fasciculus and bilateral cortical spinal fasciculus. Volume-of-interest (VOI) analyses detect the changes of diffusivity indices in the regions with FA abnormalities revealed by control vs sHDIs. In most VOIs, FA reductions were caused by the increase in Dr as well as the decrease in Da. Correlation analysis was used to assess the relationship between FA and behavioral measures in HDIs and controls available. Significantly positively correlations were found between the FA values in the right orbital-frontal WM, right parietal WM and IGT performance.

**Conclusions:**

The extent and severity of WM integrity deficits in HDIs was associated with the length of heroin dependent. Furthermore, abnormal WM microstructure may correlate with decision-making impairments in HDIs.

## Introduction

Drug addiction is a chronically relapsing brain disease, characterized by the failure to resist one’s impulses to obtain and take certain types of addictive drugs despite serious negative consequences [1]. However, the exact neurological disorders related to drug addiction are still not fully understood. A growing body of evidence has suggested that heroin addiction is a disorder of neural connectivity. Functional magnetic resonance imaging (fMRI) [2]–[4], positron emission tomography (PET) [5] and electroencephalogram [6] studies have demonstrated aberrant functional connectivity in rest [2]–[4], [6] and in active tasks such as drug craving [5], decision making [7] and inhibitory control [8] in chronic heroin dependent individuals (HDI). Given that aberrant functional connectivity might result from pathological of the white matter axonal connections, the next logical step is to understand the underlying structural architecture of the white matter (WM) in HDIs.

The neuropathologic mechanisms underlying WM changes in HDI can be investigated in vivo by magnetic resonance (MR) diffusion tensor imaging (DTI). DTI is a non-invasive MRI technique which is sensitive to the self-diffusion of water molecules. It provides measures of white matter microstructure in vivo [9]. Degradation or disruption of the microstructural organization in WM is accompanied by changes in measurable DTI parameters [10]. A number of DTI studies that compared the HDI with normal controls have been published. However, findings from these reports are remarkable inconsistent [11]–[13]. By using the region of interest (ROI) method, Wang et al did not find any differences between HDI and control groups with ROI placing in bilateral frontal lobe, temporal lobe, splenium and genu of corpus collasum [11]. By employing the whole brain voxel-based analysis methods, Liu et al demonstrated that HDI had lower FA in the white matter of the bilateral frontal sub-gyral regions, right precentral and left cingulate gyrus [12]. While Bora and his colleague found widespread FA reductions in multiple pathways including clusters spanning the CC (isthmus to genu), thalamic radiation and parietal, frontal, temporal and cerebellar tracts in HDI [13]. Although methodological differences (ROI vs. VBA vs. TBSS), sample characteristics might account for some of the bias, the different average heroin duration might also be an affect factor. In Liu et al’s study [12], the average time of heroin consumption was 5.6 years, but it was 8.9 years in Bora et al’s study [13]. In addition, both of the two studies had revealed local FA values/axial diffusivity (Da) values were negatively correlated with the duration of heroin abuse. Thus, we suppose that the duration of heroin abuse might play a vital role in the extent and severity of WM impairment in HDI.

The Iowa gambling task (IGT) is a task to assess participants’ decision making during uncertainty [14]. Decision making and impulsivity control deficits are frequently observed in HDIs in previous behavior studies and clinical observations [15]–[18]. To test whether the WM impairment relate to these behaviors aberrant, we also performed the Iowa gambling task (IGT) to assess participants’ decision making during uncertainty soon after MR imaging.

The purpose of the present study is 1) to explore the white matter microstructure changes in HDIs with different length of heroin abuse and healthy controls by using DTI and TBSS methods, and 2) to explore the association of WM differences and decision making deficits in HDI. We hypothesize that decision making deficits in HDI are associated with specific patterns of WM damage, which is dependent on heroin consumption.

## Materials and Methods

### Subjects

Fifty-two male subjects, including 16 control subjects and 36 HDI participated in this study. According to the duration of heroin use, the HDIs were divided into two groups, the sHDI (duration of heroin use <10 years) and the lHDI (duration of heroin use between 10 years and 20 years). All the HDIs were recruited from the Addiction medicine division of Guang Dong No.2 Provincial People’s Hospital, who sought for medical help on their own initiative or coerced by their family for the first time. They were screened by the Structured Clinical Interview (SCID-IV) for the Statistical Manual of Mental Disorders, Fourth Edition (DSM-IV) to confirm the diagnosis of opiate dependence according to the criteria set forth in the DSM-IV. And urine tests positive for heroin before enrolling in the treatment program. According to an interview conducted by a clinical psychologist and the laboratory report, all of the patients had never used any other types of illicit drugs. They regularly smoked cigarettes and denied any psychotropic agent a month before the fMRI scan. Inclusion criteria for the control subjects were no diagnosis of substance abuse or dependence. Exclusion criteria for all participants included neurological illness, schizophrenia or bipolar disorder, prior significant head trauma, positive HIV status, diabetes, Hepatitis C, or other major medical illness and left-handedness. This study was approved by the Research Ethics Review Board of Institute of Mental Health, the Guangdong No.2 Provincial People’s Hospital. Written informed consent was also obtained from each subject.

### Behavioral Measures

Performance data on the gambling task were available for 28 subjects [14 controls and 14 HDI (6 sHDI and 8 lHDI)]. Participants were shown four decks of cards (A, B, C and D) on a computer screen and were asked to pick a card from any of them. They were informed that each time they picked a card they would win some money as indicated by a message on the screen. Occasionally, these monetary gains were accompanied by monetary losses. The goal of the task was to maximize the monetary gain beyond the $2000 loan that participants were given to start with. Each deck (labeled A, B, C, and D) contains 60 cards. Subjects were required to make 100 choices over the testing session. Overall performance was measured by the total number of advantageous selections (C and D cards) minus the total number of disadvantageous selections (A and B cards) within blocks of 20 selections. Dividing card selections into 5 blocks of 20 allowed us to determine the rate of learning over the course of the task. Participants selected, on average, approximately 20 cards/min. This task has been shown previously to differentiate between patients with frontal cortical lesions and controls [19], [20], and drug users and controls [16].

### MRI Scanning

MRI data were obtained on a Philips 1.5-T MR scanner (Achieva Nova-Dual; Philips, Best, the Netherlands). Each subject lay supine with the head snugly fixed by a belt and foam pads. Before the DTI scan, T1-weighted 3D sequence and fluid-attenuated inversion recovery images (FLAIR) were performed to exclude subjects with abnormalities. Diffusion imaging data were acquired in 32 diffusion gradient directions (b = 800 s/mm^2^ along 32 non-collinear directions) plus a b = 0 reference images using a single shot spin echo planar sequence to collect diffusion weighted images, other imaging parameters were: TR = 10793 ms, TE = 62 ms, field of view = 230×230 mm^2^, matrix = 128×128, slice thickness = 2 mm, with no slice gap. voxel size = 2×2×2 mm^3^.

### Data Analysis

#### Data preprocessing

Before preprocessing, all the MRI images were reviewed by a neuroradiologist (YW Qiu) to exclude participants with signs of vascular accidents and other abnormalities. Then all DTI datasets were pre-processed with the FSL v4.1.7 (Functional Magnetic Resonance Imaging of the Brain Software Library; http://www.fmrib.ox.au.uk/fsl). First, the raw DTI dataset were corrected for eddy current distortion and head motion by registering the diffusion-weighted images with the null image through the affine transformations using FMRIB’s Diffusion Toolbox v2.0 (FDT, part of FSL) [21]. Subsequently, DTI dataset were skull striped using Brain Extraction Tool v2.1 (BET, part of FSL) [22] to remove background noise and non-tissue components. Then the diffusion tensor was calculated with the DTIFIT program for whole brain volumes to yield FA, mean diffusivity (MD), axial (Da) and radial (Dr) diffusivities.

#### White matter microstructure analysis (DTI-TBSS)

Resulting FA, MD, Da and Dr volumes were used in TBSS analysis. Direct registration of individual FA volumes to the FMRIB58 template was applied and as described in the original papers [23], [24], the mean FA-image was created following registration and thinned to represent the mean FA skeleton (FA>0.2 overlaid with the mean FA image). Individual FA, MD, Da and Dr volumes were then projected onto this common skeleton. Following these steps, data were fed into voxel wise cross-subject statistical analyses with following group comparisons; control vs. sHDI, control vs. lHDI and sHDI vs. lHDI. Group differences in global FA integrity were calculated by comparing mean FA values within the skeleton mask (thresholded at a mean FA value of 0.2). Nonparametric 2-sample Mann-Whitney U tests based on a permutation method were used to test for significant differences between groups because of the nonparametric distribution of the data and the number of permutation tests was set to 5000 [25]. Threshold-free cluster enhancement (TFCE) [26], an alternative to conventional cluster-based thresholding which is normally compromised by the arbitrary definition of the cluster forming threshold, was used to obtain the significant differences between two groups at p<0.05, after accounting for multiple comparisons by controlling for family-wise error (FWE) rate. From the results of voxel-wise group comparisons, the skeletal regions showing significant inter-group differences were located and labeled anatomically by mapping the FWE-corrected statistical map of p<0.05 to JHU-WM Tracto-graphy Atlas in MNI space and MNI Structural Atlas.

#### Volume-of-interest analysis of diffusion indices

In order to investigate the microstructural mechanisms of the observed FA changes, volume-of-interest (VOI) analysis was performed to access the changes of diffusivity indices (Da,Dr) in the regions showing FA abnormalities between the sHDI and controls in all HDIs. To do so, the VOI masks were firstly extracted based on the clusters showing significant inter-group FA differences. These VOIs masks were then back-projected to the original images of each subject, and the mean values of the diffusion indices within the VOIs were calculated. After confirming normal distribution of the data by a one-sample Kolmogorov-Smirnov test, one-way ANOVA was performed to detect the differences among the groups. A statistical significance level of p<0.05 (Bonferroni correction for multiple comparisons) was used. Pearson correlation analyses were used to test the correlations between FA changes within the VOIs and behavioral measures, and the duration of heroin use. A p<0.05 was considered statistically significant.

## Results

### Demographic and Behavioral Measures

In the 18 sHDI, 6 of them inhaling the vapor from heated heroin, 7 of them using intravenous, and 5 of them with both methods. In the 18 lHDIs, 7 with inhaling, 7 with intravenous, and 4 with both methods. There was no significant difference in age, education, number of cigarettes smoked per day among sHDIs, lHDIs and control subjects. No significant difference exists in average heroin consumption per day between sHDIs and lHDIs. The lHDIs consumed heroin much longer than the sHDIs (P = 0.000). (Table 1).

**Table 1:** Subject demographics for heroin-dependent individuals (HDI) and control groups.

Items	Controls (n = 16)	sHDI (n = 18)	lHDI (n = 18)	Group Effect	
				F(t)	P
Age (years)	37.56±4.273	35.11±8.443	37.89±4.213	1.632	0.206
Education (years)	9.94±3.838	9.50±2.572	8.44±3.502	1.123	0.333
Cigarette (no. of cigarettes/d)	20.94±10.036	17.22±8.440	15.56±8.024	0.920	0.405
Duration of heroin use (years)	N/A	5.665±3.0339	16.111±2.9082	−10.546	0.000*
Mean dosage (g/d)	N/A	0.67±0.55	0.62±0.34	0.330	0.743
IGT score	6.29±6.603 (14)	−1.67±4.274 (6)	−4.75±6.409 (8)	9.162	0.001*

* Significant different, P<0.05.

sHDI: short-term dependence, heroin dependent individuals with duration less than 10 years;

lHDI: long-term dependence, heroin dependent individuals with duration between 10 to 20 years;

IGT: The Iowa gambling task.

**Figure 1:**
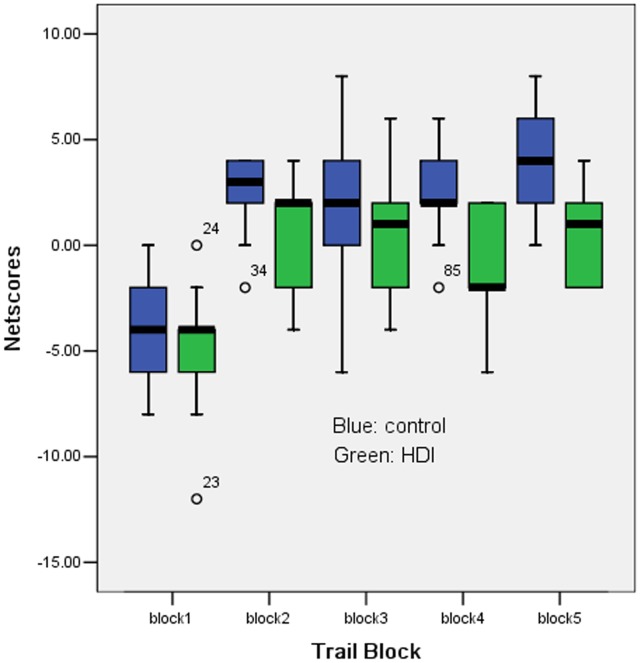
Performance on Iowa Gambling Task (IGT). All participants increased net score over time consistent with learning. Controls had higher net scores than HDI.

The IGT performance was analyzed by a repeated analysis of variance model with validated sphericity assumption (P = 0.741). A significant main effect of IGT trial block (P<0.001) was observed, with both groups selecting significantly more cards from the bad decks during the early trials of the task and progressively selecting more cards from the good decks during the later trial blocks. Most important, there was a significant main effect of group (P<0.001), indicating that the HDI group made significantly more selections from the bad decks than the control group (Fig. 1). Covariates age (P = 0.895), education (P = 0.212), and number of cigarettes smoked (P = 0.446) had no significant effect on IGT performance.

### TBSS Results

**Figure 2:**
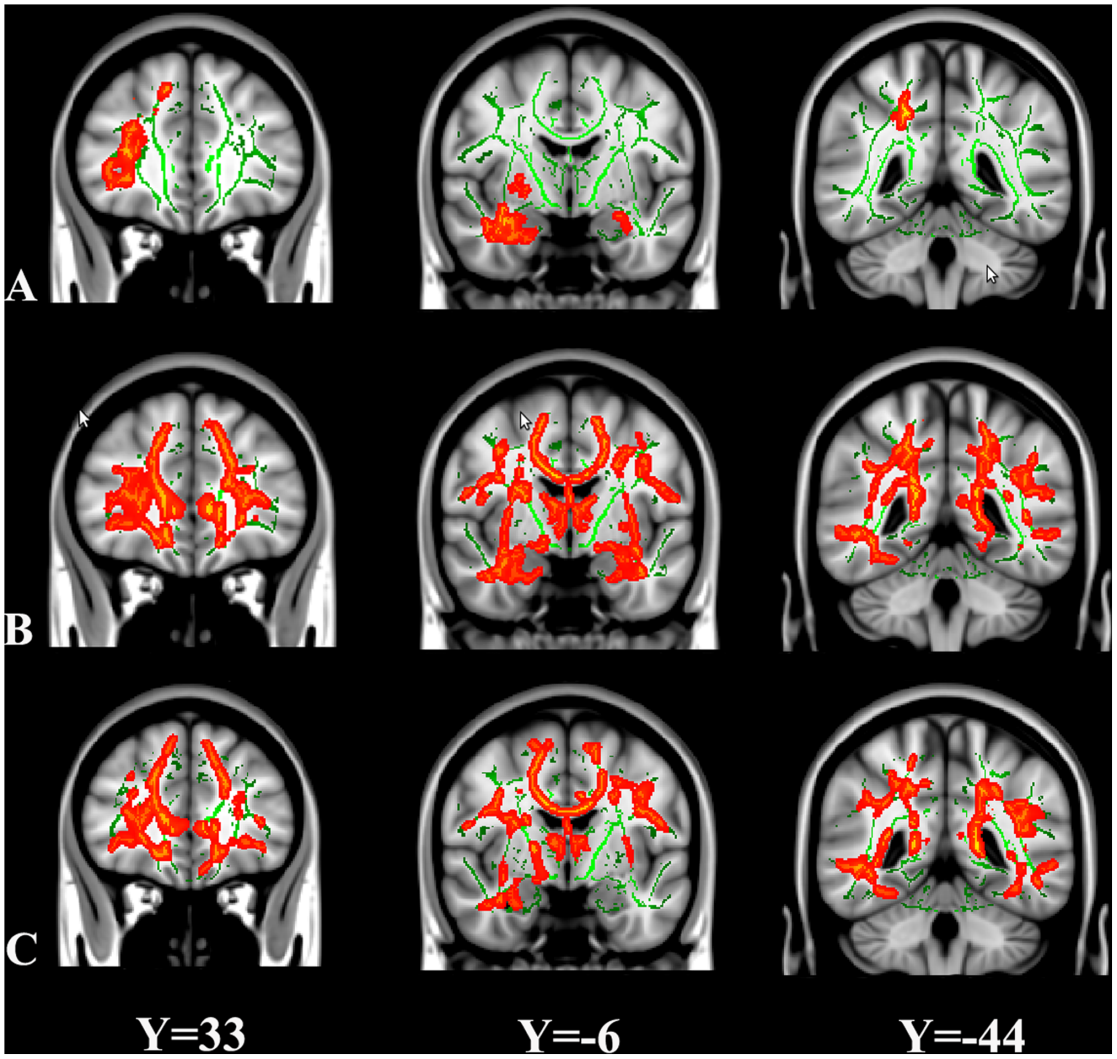
TBSS analysis of fractional anisotropy (FA) volumes. Areas in red are regions where FA was significantly lower (p<0.05, corrected by TFCE) in sHDI (Panel A) and lHDI (Panel B) relative to normal controls, and in lHDI (Panel C) relative to sHDI. To aid visualization, regions showing reduced FA (red) are thickened using the tbss_fill script implemented in FSL. Results are shown overlaid on the MNI152-T1 template and the mean FA skeleton (green). The left side of the image corresponds to the right hemisphere of the brain.

**Figure 3:**
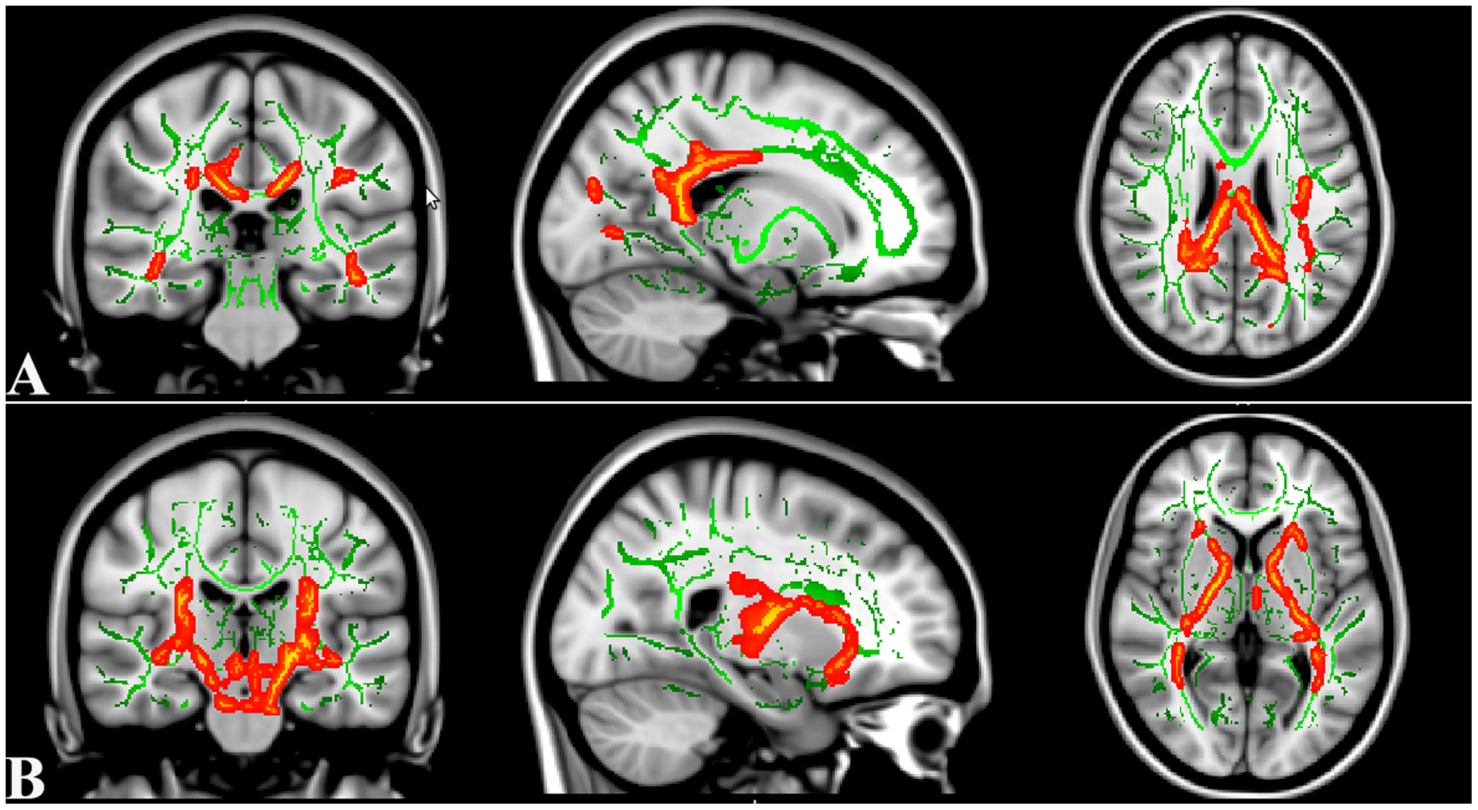
TBSS analysis of axonal diffusivity (Da) and radial diffusivity (Dr) volumes. Compared with controls, the lHDI showed significantly reduced Da in bilateral inferior frontaloccipital fasciculus, bilateral splenium of corpus callosum, and left inferior longitudinal fasciculus (Panel A), and showed significantly increased Dr in bilateral uncinatus fasciculus, bilateral inferior frontaloccipital fasciculus and bilateral cortical spinal fasciculus (Panel B). To aid visualization, regions showing reduced Da (red) or increased Dr (red) are thickened using the tbss_fill script implemented in FSL. Results are shown overlaid on the MNI152-T1 template and the mean FA skeleton (green). The left side of the image corresponds to the right hemisphere of the brain.

A value of 0.2 was used to threshold the mean FA skeleton volume. Figure 2 showed the spatial distribution of the brain regions indicating a reduction of FA in the sHDIs group when compared with controls, and in the lHDIs when compared controls and sHDI. Compared to the control subjects, sHDI subjects had significantly reduced FA (P<0.05; TFCE-corrected) in right orbito-frontal white matter (WM), bilateral temporal WM and right parietal WM. The lHDIs subjects displayed widespread FA reductions, which included clusters spanning the CC (isthmus to genu), thalamic radiation and parietal, frontal, and temporal tracts. Despite the range is not as extensive as lHDI vs. control group, the lHDIs subjects showed widespread FA reductions compared with sHDIs subjects, including clusters spanning the CC (isthmus to genu), thalamic radiation and parietal, frontal, and temporal tracts (Fig. 2). Neither white matter regions in which the controls had significantly lower FA values than the HDIs groups, nor the sHDIs compared with the lHDI. Figure 3 showed the spatial distribution of the brain regions indicating a reduction of Da and increased of Dr in the lHDIs group when compared with controls. The lHDI groups had significantly lower Da than controls in bilateral inferior frontaloccipital fasciculus, bilateral splenium of corpus callosum, and left inferior longitudinal fasciculus, and had significantly higher Dr value than controls in bilateral uncinatus fasciculus, bilateral inferior frontaloccipital fasciculus and bilateral cortical spinal fasciculus (Fig. 3). No significant differences were found between control vs. lHDI in MD value, nor any significant difference between control vs. sHDI and sHDI vs. lHDI in MD, Da, and Dr values.

### VOI Results

**Figure 4:**
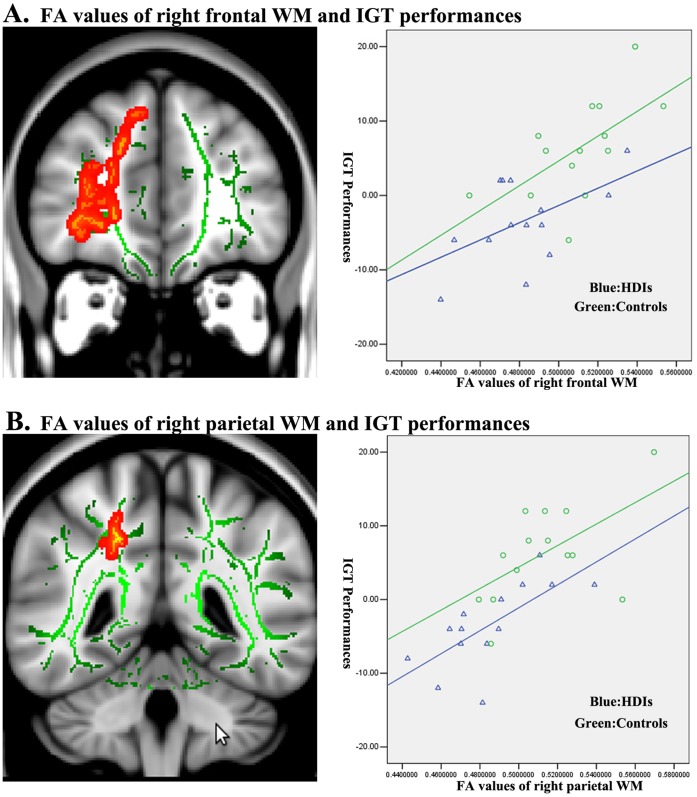
Correlation analysis of the FA values and the IGT performance. The FA values of the right frontal WM, right parietal WM were positively correlated with the IGT performance in both HDI and controls.

The 4 brain regions with significantly reduced FA in the sHDIs group were extracted for VOI-based analysis of other diffusion indices. The results are listed in Table 2. All the four VOIs (except for the right orbital-frontal WM with increased Dr only) showed significantly increased Dr and reduced Da (P<0.05, Bonferroni correction for 3 comparisons). For the 4 VOIs, Pearson correlation analysis demonstrated significantly positive correlations between IGT performance in all HDIs available and FA values in the right frontal (r = 0.533, P = 0.049), right parietal (r = 0.707, P = 0.005) individually (Fig. 4). There were significantly negative correlations between duration of heroin dependent in all HDIs and FA values of the right frontal (r = −0.344, P = 0.040), right parietal (r = −0.489, P = 0.006), and right temporal (r = −0.397, P = 0.036) (Table 3).

**Table 2:** Group differences in diffusivity indices from volume-of-interests.

Anatomic region	Diffusivityindices	controls	sHDIs	lHDIs	Group EffectF_(2, 51)_ (P )	Post hoc
Right frontal WM	Da	1.433×10^−3^±3.067×10^−5^	1.427×10^−3^±3.129×10^−5^	1.415×10^−3^±3.810×10^−5^	1.283(0.286)	
Right frontal WM	Dr	6.381×10^−4^±2.382×10^−5^	6.524×10^−4^±2.790×10^−5^	6.670×10^−4^±2.722×10^−5^	5.032(0.010*)	0.003#
Right parietal WM	Da	1.427×10^−3^±3.328×10^−5^	1.393×10^−3^±3.065×10^−5^	1.266×10^−3^±3.890×10^−5^	18.765(0.000*)	0.000^#$^
Right parietal WM	Dr	6.016×10^−4^±5.166×10^−5^	6.854×10^−4^±4.267×10^−5^	6.855×10^−4^±4.701×10^−5^	4.619(0.015*)	0.011^#$^
Right temporal WM	Da	1.421×10^−3^±3.636×10^−5^	1.417×10^−3^±2.819×10^−5^	1.189×10^−3^±1.333×10^−4^	47.331(0.000*)	0.000^#$^
Right temporal WM	Dr	6.379×10^−4^±5.367×10^−5^	6.740×10^−4^±4.746×10^−5^	7.001×10^−4^±5.206×10^−5^	6.348(0.004*)	0.044$ 0.001#
left temporal WM	Da	1.437×10^−3^±3.077×10^−5^	1.411×10^−3^±3.958×10^−5^	1.406×10^−3^±2.946×10^−5^	4.241(0.020*)	0.028$ 0.008#
left temporal WM	Dr	6.369×10^−4^±4.872×10^−5^	6.743×10^−4^±3.178×10^−5^	7.130×10^−4^±6.276×10^−5^	10.042(0.000*)	0.033$ 0.000# 0.023&

* Significant different, P<0.05.

$ Significant different for control vs sHDIs.

# Significant different for control vs lHDIs.

& Significant different for of sHDIs vs lHDIs.

WM: white matter; Da: axial diffusivity; Dr: radial diffusivity.

**Table 3:** Correlation between mean FA of Abnormal Brain Regions (revealed by sHDI vs controls) and Duration of Heroin Use in HDIs.

Anatomic region	Diffusivity indices	MNI coordinate(mm) (x,y,z)	Correlation Coefficient	P values
Right Frontal WM	FA	30,40,10	−0.344	0.040*
Right Frontal WM	Da	30,40,10	−0.257	0.154
Right Frontal WM	Dr	30,40,10	0.158	0.365
Right parietal WM	FA	20, −46,42	−0.489	0.006*
Right parietal WM	Da	20, −46,42	−0.423	0.010*
Right parietal WM	Dr	20, −46,42	0.167	0.357
Right temporal WM	FA	32, −5, −23	−0.542	0.001*
Right temporal WM	Da	32, −5, −23	−0.113	0.512
Right temporal WM	Dr	32, −5, −23	0.302	0.073
left temporal WM	FA	−28, −11, −24	−0.312	0.064
left temporal WM	Da	−28, −11, −24	−0.117	0.495
left temporal WM	Dr	−28, −11, −24	0.341	0.042*

* P<0.05.

## Discussion

Our findings indicated that the extent and severity of white matter integrity deficits in HDIs were associated with heroin duration. The longer the subjects consumed heroin, the more widespread and serious the white matter integrity was disrupted. Furthermore, the disruption of white matter integrity in HDIs seems related to both the myelin pathology and axonal injury, given the findings of increases in radial diffusivity and decreases in radial diffusivity [27], [28]. Furthermore, we also discovered the relationship between impaired decision making and FA values in right frontal and parietal WM regions in HDIs. Thus, the disrupted white matter microstructure in these regions might be the neural mechanism of decision-making deficits in HDIs. This data complement our previous BOLD fMRI-based study, which showed that impaired IGT performance was related to reduce regional homogeneity in bilateral medial OFC in HDIs [29].

The sHDIs group, with average 5.7 years heroin consumption, showed local white matter integrity deficits, mainly located in right frontal, right temporal and right parietal WM regions, which was consistent with Liu et al’s study [12], with average 5.6 years (68 months) heroin consumption, their HDIs group also showed local white matter integrity deficits, and these deficits mainly located in the bilateral frontal sub-gyral regions, right precentral and left cingulate gyrus. While the lHDIs group (average heroin consumption is 16.1 years) showed diffuse white matter integrity deficits, including clusters spanning the CC (isthmus to genu), thalamic radiation and parietal, frontal and temporal tracts, which agreed with Bora and his colleague study [13], with an average of 8.9 years heroin consumption, their HDIs groups also showed diffuse white matter integrity deficits. This finding suggested that the extent of WM impairment was related to the length of heroin abuse, the longer duration the HDIs consumed heroin, and the more widespread the brain white matter was involved. This finding partially addressed the discrepancies of previous DTI studies in heroin addiction mentioned above, and also supported our hypothesis that the duration of heroin consumption may play a vital role in the extent of white matter integrity deficits.

We also observed a negative correlation between the FA values in right orbital-frontal, right temporal, and right parietal sub-gyral regions and the duration of heroin use in all HDIs, which meant the severity of local white matter integrity deficits was associated with the duration of heroin consumption. This observation complemented previous functional [29] and structural [30] neuroimaging studies, which found out that the brain functional as well as the gray matter volume/density was progressively damaged along with the duration of drug abuse in HDI. It also agreeed with behavior studies that heavier use of opiates in long-term users was associated with greater likelihood of neuropsychological impairment [31]. Together with previous findings, it was plausible for us to demonstrate heroin addicts could impair the gray matter volume/density and white matter microstructure progressively, which may underpin the functional deficits in HDI.

The VOI analysis indicated that heroin related white matter micro-structural damage in HDIs group was manifested as reduced axial diffusivity and increased radial diffusivity in right parietal and right temporal WM, while increased radial diffusivity with no significant change in axial diffusivity in right frontal WM. This finding suggested that reduced axial diffusivity combined with increased radial diffusivity played a role in the WM impairment in HDI, and myelin dissolution (affecting radial diffusivity) dominated these changes. This finding was inconsistent with Bora reports, which demonstrated the heroin addicts were associated with widespread deficits in white matter integrity with myelin pathology only, but with not axonal injury. Several factors might explain these differences. First, in Bora et al’s study, some of their HDIs also abused other addictive or psychoactive drugs (cannabis and benzodiazepines), which was apparently known to be the cause of brain white matter changes [32]. Thus their findings may be a combinative effect of heroin and cannabis on the brain. However, none of our subjects was other addictive or psychoactive drugs dependent, thus the results of the present study were more relevant to the heroin than the previous study. Second, differences in severity of heroin use might play a role. The average heroin consumption (16.1 years) of HDI in our lHDI groups was longer than that of Bora et al’s study (8.9 years). In Bora et al’s study, they also found a negative correlation between the axial diffusivity and duration of heroin use in their HDI, especially for the longitudinal fasciculus and the right frontal lobe white matter. And given the evidence of increased ischemic lesions in HDIs [33], the observed diffusion abnormalities might result from perfusion deficits and/or ischemia related to respiratory suppression, overdoses, disturbance of consciousness, vasculitis and rhabdomyolosis in HDIs, which related to both axonal disrupted and myelin pathology [27]. The axonal injury in HDI observed in present study was also consistent with previous autopsy study in heroin predominant poly-drug addicts. Through immunohistochemistry analysis, Büttner and his colleague found β-APP-immunopositive accumulations were increased significantly in white matter of the patients compared with controls, which indicated diffuse axonal damage [34].

Interestingly, we also observed the positive correlation between the FA values in right orbital-frontal and parietal WM and the IGT performance in both HDIs and control groups. Given the right orbital-frontal, right parietal WM is the part of frontal-parietal network. These findings supported the idea that the IGT performance related with the frontal-parietal brain network [35]–[37]. In turn, it also suggested that the FA values could serve as a reliable biomarker when exploring the white matter integrity. Thus, FA might be as a qualified biomarker to understand the underlying neural mechanisms of injury or to assess the effectiveness of specific early interventions in HDI.

Some limitations in our study were worth mentioning. First, the assessment of small fiber tracts was difficult with TBSS, because only the major white matter tracts were included in the skeleton [23]. Second, because of the nonlinear alignment process, FA, Dr and Da values that were attributed to a given voxel were not actually measured at that spatial location but pulled in from a neighborhood, which may disguise the results [23]. Third, our DT imaging sequence had only one acquisition at b equals 0 sec/mm^2^ as a limitation of the imager, which made the DT imaging acquisitions scheme suboptimal [24]. Last, as chronic heroin dependence may be complicated by depression, and recent study had found that depression itself can disrupt the white matter microstructural [38]. Thus lack of co-morbid depression assessment is another limitation of this study, a more rigorous experiment is needed to exclude the influence of depression in the future study.

### Conclusion

Our findings indicated that the extent and severity of white matter integrity was progressively disrupted in HDIs. Furthermore, the abnormal white matter integrity might relate to decision-making impairments in HDIs. Thus, FA, index of white mater integrity, may be as a qualified biomarker to understand the underlying neural mechanisms of injury in HDI.
